# Does women’s age influence zona pellucida birefringence of metaphase ΙΙ oocytes in in-vitro maturation program?

**Published:** 2013-10

**Authors:** Marjan Omidi, Mohammad Ali Khalili, Hossein Nahangi, Sareh Ashourzadeh, Marzieh Rahimipour

**Affiliations:** 1 *Department of Biology and Anatomical Sciences, Shahid Sadoughi University of Medical Sciences, Yazd, Iran.*; 2 *Research and Clinical Center for Infertility, Shahid Sadoughi University of Medical Sciences, Yazd, Iran.*

**Keywords:** *ZPbirefringence*, *Morphology*, *Humanoocytes*, *Female age*.

## Abstract

**Background:** In vitro maturation (IVM) is a promising treatment option for certain infertile women. Nowadays, with the aid of PolScope, it has become possible to evaluate zona pellucida (ZP) characteristics as a parameter of oocyte quality. Moreover, quality of oocytes can be influenced by many factors, such as patient’s age. The PolScope system is a non-invasive technique to assess birefringent structures such as the meiotic spindle and ZP in living oocytes.

**Objective: **The aim was to determine the influence of the woman's age on ZP birefringence, a sign of oocyte quality, and morphology of in-vitro matured human oocytes using non-invasive polarized light (PolScope) microscopy.

**Materials and Methods:** ZP birefringence and morphology were determined in 105 retrieved oocytes from 58 women undergoing ICSI in two age groups (≥30 years and <30 years). The immature oocytes were selected and after IVM, the quality of metaphase ΙΙ (MII) oocytes was assessed. The oocytes abnormalities were classified as intracytoplasmic and extracytoplasmic abnormalities.

**Results: **Oocyte maturation rates were significantly reduced in ≥30 year’s women (56%) in comparison with other age group (80.7%). In addition, the ZP birefringence was significantly higher in MII oocytes in the younger group compared with the older group (76.2% vs. 38.1%; p=0.00). Following morphologic assessment, the rates of oocytes with extracytoplasmic (p=0.02) and both abnormalities (extra- and intracytoplasmic) (p=0.01) were higher in aged versus the younger women.

**Conclusion:** There was a positive relationship between advanced maternal age with decreased ZP birefringence and oocyte morphological quality in in-vitro matured human oocytes.

## Introduction

Introduction of ICSI was a grand achievement to give the chance of having babies for infertile couples. Following denudation in ICSI, oocytes can be assessed in more details and accurate manner based on the morphology of the ooplasm and on the aspect of the extracytoplasmic structures such as the ZP, first polar body (1PB) and perivitelline space (PVS) (1, 2). Since, the objective of morphological assessment is one of the main prognostic factors of oocyte quality, morphological evaluation before ICSI helps to recognize oocytes with higher developmental potential (3, 4). Studies have shown that 15-20% of the oocytes remain immature at the time of oocyte retrieval (5). In vitro maturation (IVM) of such immature oocytes can be a way to increase the number of embryos, although pregnancy and implantation rates have been reported very rare in IVM program (6). 

The influence of MΙΙ oocyte quality on the outcome of assisted reproduction technology (ART) programs has been very controversial. Some investigators reported that the better rates of fertilization and embryo development in ART cycles is correlated with oocyte normal morphology (-). Others, however, have demonstrated that oocyte morphology does not affect fertilization rate, embryo quality and implantation rate after ICSI (10, 11). Gross morphologic alternations of MΙΙ oocytes include non-spherical shape of oocyte, ooplasm granularity, smooth endoplasmic reticulum cluster (SERc), refractile body (RF), bull eye, wide PVS, debris in the PVS, vacuolization, and abnormalities of 1PB or ZP (6). 

New methodologies, such as polarization light microscopy have been proposed as methods to advance the objective choice of high quality oocytes. With the aid of PolScope, it has become possible to evaluate meiotic spindle and ZP characteristics due to their natural birefringence, noninvasively in live human oocytes (12, 13). The ZP is a multilaminar glycoprotein coat composed of filaments organized in different orientation surrounding the maturing oocytes during ovulation up to embryonic development (14). In particular, ZP appears to be divided into two birefringent layers separated by an anisotropic layer (15). Software programs are currently available that automatically analyze the ZP birefringence scores based on the intensity and distribution of the birefringence (12).

In addition, several factors, including women age affect the quality of oocyte. This condition has been associated with decreased fertility potential and poor oocyte quality (16, 17). The aim of this prospective study was to evaluate the influence of patient’s age on morphologic variables and ZP birefringence score of human oocytes undergoing IVM. 

## Materials and methods

**Study design**

This cross-sectional study included MΙΙ oocytes (n=105) obtained after IVM from 58 women (21-38 years, mean age±SD: 28.9±4.5 years) who were admitted to ICSI program at the Research and Clinical Center for Infertility (Yazd, Iran). A total of 689 oocyte-cumulus complexes (OCC) were retrieved, out of which 105 (15.2%) were at MΙΙ after IVM for 24-40 h. The present study was approved by Ethics Committee of our institution Research and Clinical Center for Infertility (Yazd, Iran). 

Oocytes were classified according to the age of women (younger, <30; older, ≥30years). The oocytes were also categorized according to their ZP birefringence into high and low ZP birefringence (HZB and LZB). Then, the influence of the female patient age on the ZP birefringence was evaluated. Moreover, the effect of maternal age on the morphological parameters of oocytes was assessed.

**Ovarian stimulation, oocyte retrieval, preparation and maturation**

Patients were stimulated with exogenous GnRH-agonist or antagonist and follicle stimulating hormone (FSH; Ferring Co, Germany). Monitoring of follicular development was performed by ultrasonography. The recombinant hCG (rhCG; IBSA Co, Switzerland) was administered when the ovarian follicles reached 18-20 mm diameter, followed 36 h later by oocyte retrieval. 

2-3h after oocyte collection, following incubation of 30-60 s exposing to 80 IU/mL hyaluronidase (Irvine Scientific, USA), oocytes were treated by pipetting to remove the cumulus cells. The denuded oocytes were then assessed for nuclear status. Based on extrusion of 1PB, oocytes were considered MΙΙ or immature (GV or MΙ). Immature oocytes were cultured in maturation medium (SAGE, USA) supplemented with 75 mIU/mL FSH and 75 mIU/mL LH (Ferring, Germany) at 37^o^C in incubator with 5% CO_2_ and 95% air with high humidity (18).

**Screening of oocytes and live ZP birefringence analysis**

For ZP birefringence evaluation, each mature oocyte was placed in a 3 µL droplet of buffered medium (G-Mops-V1; vitrolife, Sweden) in a glass bottomed culture dish (WillCo- Dish) covered with warm mineral oil (Irvine Scientific, USA). ZP imaging was performed non-invasively on Nikon TE-300 inverted microscope. The images were captured and saved for evaluation of morphologic variables. 

**Figure 1:**
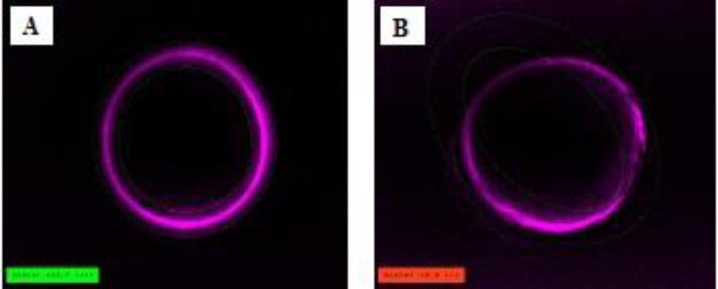
The inner layer of zona pellucida of MΙΙ oocyte imaged with the Polscope. A) high ZP birefringent oocyte B) low ZP birefringent oocyte. ×40 eyepiece magnification

The birefringence analysis, including autocalibration, was controlled by a polarization imaging software module (OCTAX ICSI Guard™, Microscience, Germany) implemented with an imaging software system (OCTAX Eyeware™). In particular, the software calculated a score according to concentration and homogeny around the entire cell; oocytes with a birefringence score ≥10 were considered as HZB and with a <10 score as LZB (Figure 1) (18).

**Oocyte morphology assessment**

The captured images were used to evaluate morphologic parameters. The oocytes morphologic characteristics were categorized according to the presence of intracytoplasmic or extracytoplasmic abnormalities. Intracytoplasmic abnormalities were determined by variables of irregular shape, vacuole, RF, SERc, ooplasm granulation, and bull eye. Extracytoplasmic abnormalities included wide PVS, PVS debris, and fragmented 1PB (7, 8). 

**Statistical analysis**

Data were presented as mean±SD. Moreover, data were reported as odds ratio (OR), 95% confidence interval (95% CI). The results were compared by chi-square and fisher’s exact tests. Data analysis was performed using SPSS (version 18). Differences were considered statistically significant at p˂0.05.

## Results

A total of 689 oocytes were retrieved from ICSI cases. Based on the presence of a 1PB, 514 of the 689 (74%) oocytes were classified as MΙΙ; 153 (22%) displayed no PB and were identified as GV or MΙ. A total of 22 (3%) oocytes were discarded because of their abnormal morphological appearance. In this prospective study, the immature oocytes were used for IVM technology. After IVM, 105 (68.6%) of the immature oocytes extruded the 1PB and reached to MΙΙ stage. A total of 105 in-vitro matured oocytes from 58 patients were studied. The numbers of immature oocytes were 78 in younger and 75 in older women. In sub-analysis, the oocyte maturation rate was 56% (42/75) in ≥30 year’s women. 

This was significantly lower than the immature oocytes matured in vitro in ˂ 30 year’s women which was 80.7% (63/78) (p<0.001). The analysis showed that the percentage of LZB oocytes was higher in older group than younger group (61.9% vs. 23.8%; p*=*0.00) (Table I). Also, the data demonstrated that HZB oocytes were significantly more numerous in younger women (Mean±SD of age in HZB vs. LZB: 29.7±0.4 vs. 31.5±0.5; p*=*0.035). 

There was no significant correlation between each oocyte abnormality and age, except for 1PB, which was higher in older women (p*=*0.02, Table II). In sub-analysis, the oocytes with extracytoplasmic and both extra- and intracytoplasmic abnormalities were significantly directly related to the women’s age (Table III).

**Table Ι:** The correlation of women’s age and oocyte ZP birefringence

**ZP birefringence**		**HZB**		**LZB**		**Odds ratio(95%CI)**	**p-value** *
		**n**	**%**	**n**	**%**		
Women’s age (years)						5.2(2.22-12.17)	0.00
	˂ 30	48	76.2	15	23.8		
	≥ 30	16	38.1	26	61.9		

HZB: high ZP birefringence.

LZB: low ZP birefringence.

* Chi-square test.

**Table ΙΙ:** The comparisons of morphological parameters between oocytes from older and younger women

** Women age **		**˂30 years**	**≥30 years**	**Odds ratio (95% CI)**	**p-value** *
**Parameters**					
Intracytoplasmic abnormality					
	Irregular shape	39.7	28.6	1.65(0.71-3.80)	0.29
	Vacuole	19	31	0.52(0.21-1.30)	0.17
	Refractile body	27	40.5	0.54(0.23-1.24)	0.20
	SERc	12.7	2.4	5.96(0.71-49.5)	0.08
	Bull eye Ooplasm granulation	7.9 12.7	11.9 19	0.63(0.17-2.35) 0.61(0.21-1.80)	0.51 0.41
Extracytoplasmic abnormality					
	Wide PVS	34.9	47.6	0.59(0.26-1.30)	0.22
	PVS debris	14.3	21.4	0.61(0.21-1.69)	0.43
	Fragmented PB	25.4	47.6	0.37(0.16-0.85)	0.02

Values represent percent (%).

* Chi-square test.

**Table ΙΙΙ:** Correlation between oocyte morphology and female age

** Women age**	**˂30 years**	**≥30 years**	**Odds ratio (95% CI)**	**p-value** *
**Parameters**				
ICA	77.8	81	0.82 (0.31-2.17)	0.80
ECA	55.6	78.6	0.34 (0.14-0.82)	0.02
Both AB	42.9	66.7	0.37 (0.16-0.84)	0.01
No AB	11.1	7.1	0.82 (0.31-2.17)	0.73

ICA: intracytoplasmic abnormality.

ECA: extracytoplasmic abnormality.

AB: abnormality.

Values represent percent (%).

* Chi-square test.

## Discussion

Both nuclear and cytoplasmic maturations and synchrony of them determine the quality of oocyte (3). The morphology of oocytes may have a direct effect on the reproductive success in ART cycle (19-20). Numerous criteria have been existed to recognize the best oocytes, in regards to morphological, cellular, and molecular aspects (19).Until now, only a few predictive noninvasive markers for oocyte quality have been identified on the basis of morphologic criteria, which can be assessed using conventional microscopy (21). The introduction of polarization light microscopy has enabled the noninvasive visualization of subcellular structures in oocytes, such as the structure of ZP. 

Polarized microscopy was demonstrated to be highly predictive of human oocyte quality (22). Many studies noticed a positive correlation between high morphological quality of oocytes and the major reproductive indices such as fertilization, embryo implantation and pregnancy rates (6, 13). In order to have a complete morphological view of oocytes, transmission electron microscopy (TEM) evaluation is especially effective in estimating the oocyte structural integrity (23). Although, it is costly, time consuming, and requires oocyte fixation, which makes it unusable for clinical application. Several recent studies indicated that properties of the ZP layers might reflect the history of oocyte cytoplasmic maturation (24, 25). 

It has been proposed that different development stages and culture conditions may alter the ZP architecture of human oocytes (23). Braga *et al* suggested that ZP birefringence decreases as oocyte in vivo nuclear maturation takes place (25). However, they proved that during IVM protocol, ZP birefringence remains unaffected. Recently, in another study we assessed the presence of meiotic spindle and ZP birefringence in both in-vivo matured and IVM oocytes. Our findings suggested that clinical IVM is a safe technology that maintains the high maturation rate and integrity of oocytes (18). 

Recently, ultrastructural analysis revealed an increasing morphological abnormality after IVM technique, as the major abnormality was related to numerous large mitochondria-vesicle complexes in oocytes compared with in-vivo matured oocytes (23, 26). Our finding showed that 22% of retrieved oocytes were immature, which is slightly higher than other findings (5). Also the results generated from this study declared that the rates of maturation was reduced in aged women whereas, Mohsenzadeh *et al* reported that there was no significant relation between patient's age and the rates of oocyte maturation (27). It should be noted that the IVM medium which was used in this experiment was different than previous reports. 

Our IVM medium was commercial, whereas they applied the home-made medium for IVM of human oocytes (5, 27). The basal IVM rate of GV stage oocytes collected from stimulated cycles differ widely between studies. Differing reports of IVM rates are probably due to several factors, including IVM medium, the source of oocytes (unstimulated vs. stimulated cycles) and whether or not cumulus cells are retained with the oocyte (3).

Furthermore, the data showed that the rate of maturation was lower in GV stage than MΙ stage. Considering that nuclear maturation consists of the GV breakdown, the resumption of the meiosis, and the 1PB extrusion, it is hypothesized that the exposure to in-vitro environment during a more complex phase of development may have important consequences for the potential of human oocytes (24). In the literature, the influence of age on the fertilization rate is controversial. There are indications of both a reduced fertilization rate as well as an uninfluenced impact by age and FSH values (28, 29). 

Also findings reported the adverse impacts of age on the success rates of ART cycles (30). In the present paper, we assessed the correlation of patient’s age and indices of oocyte quality, morphologic parameters and ZP birefringence, in IVM cycles. Halvaei *et al* detected that the chance of MI oocyte retrieval is increased in older women (6). One reason may be related to ovarian function which is decreased with advancing of age as well as the reduction of ovarian response to hyperstimulation. We observed that HZB inversely correlated with patient’s age. The above results indicated the poor effects of age on the ZP birefringence. 

The role of oocyte aging as one of the most important factors in the failure of the ARTs was verified. Oocyte aging has been associated with several morphological alternations, including changes in structure of the plasma membrane, ZP, displacement of 1PB and cortical granules (30). The relationship between ZP structure and aging is controversial. Parallel with our findings, Valeri *et al *showed that there is a significant inverse correlation between ZP birefringence and women’s age (20). They also demonstrated a positive correlation between thickness of the ZP and its birefringence. Their data showed a reduced ZP thickness in older women, whilst some studies have suggested straight correlation between ZP thickness and patients age (31). 

The reduction of ZP thickness can be a consequence of a decreased ability by granulosa cells and aged oocyte to synthesize and assemble ZP proteins. Actually, defects in the synthesis and secretion of ZP proteins trigger to thinner and minimized structured ZP (32). It is known that advanced female age is well correlated with the poor quality of oocytes. Our data showed that the rates of extracytoplasmic abnormalities were increased in older women. Although, we did not notice any significant differences between rates of each oocyte dysmorphism in two groups, except for fragmented 1PB.

Khalili *et al *declared that age factor is probably responsible for the higher rates of morphological abnormalities of oocytes in women over the age of 30 years (7). One study detected that there was no significant differences for intracytoplasmic abnormality between young and old women (6). Also, it is proposed that the extracytoplasmic abnormalities (e.g. fragmented 1PB) should be considered only a phenotypic alternation of the oocytes (33). So, advanced maternal age could be a risk factor for these abnormalities. On the other hand, we cultured the oocytes in vitro that the conditions of maturation could influence on morphologic features and this may explain the apparently conflicting findings.

## Conclusion

In conclusion, The ZP birefringence and morphological quality were lowered in oocytes matured after IVM from the older women undergoing ICSI. Therefore, advanced maternal age is a risk factor for poor quality of human oocytes, which possibly affect the ART outcomes.

## Conflict of interest

There was no conflict of interest regarding our results.
